# Supplementary material for: Potassium channel-based optogenetic silencing
Source: Nat Commun. 2018 Nov 5;9:4611. doi: 10.1038/s41467-018-07038-8 (PMC6218482; doi:10.1038/s41467-018-07038-8)
Supplement: Supplementary file 1 — Supplementary Information [file 41467_2018_7038_MOESM1_ESM.pdf]

Supplementary Information

Potassium channel-based optogenetic silencing

Bernal-Sierra[+], Rost[+], Pofahl[+] et al.

Supplementary Figs. 1-5 and Supplementary Table 1 and 2

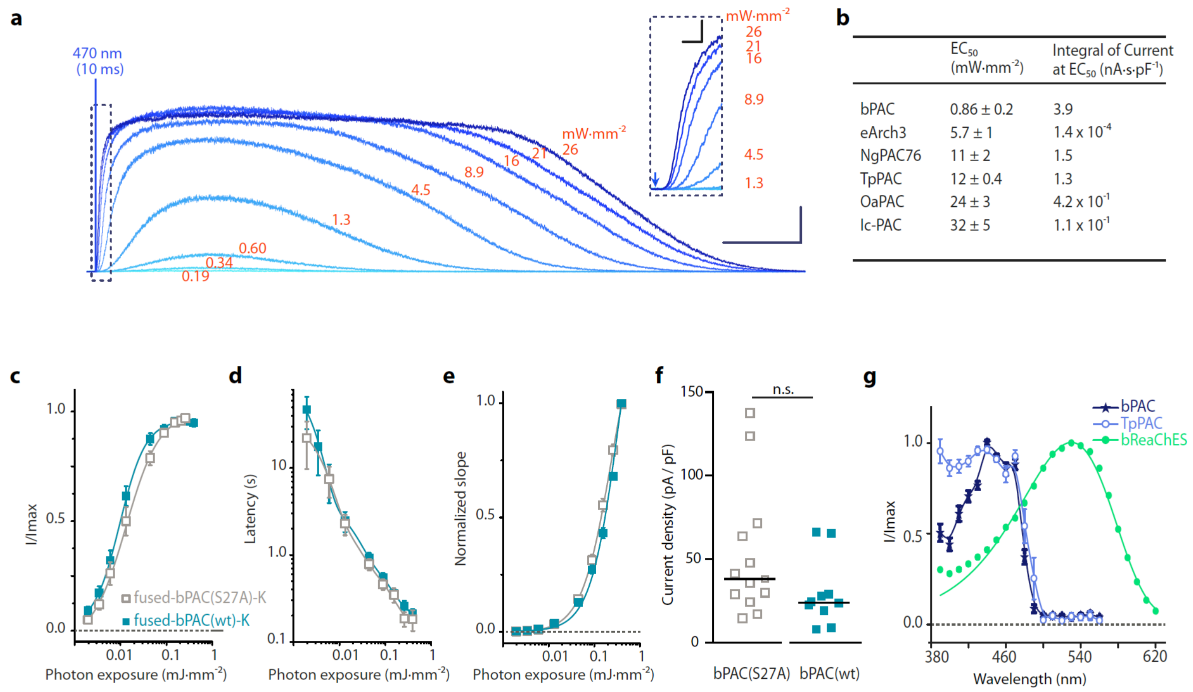

Supplementary Fig. 1: bPAC-SthK photocurrent properties in ND7/23 cells.
a Split-bPAC-K photocurrent traces elicited by a 10-ms light pulse at 470 nm and increasing light intensities. Inset shows zoom-in of the traces at current onset. Scale bars: 10 s, 500 pA (overview trace); 0.5 s, 250 pA (inset). **b** Photocurrent integrals of different Split-PAC-K constructs and Arch3. Current integrals were calculated at EC_{50} using the logistic fit equation of dose-response curves. **c-f** Comparison of photocurrent properties of SthK fused to bPAC wild type (bPAC(wt)-K)(n = 10, 3 cultures) or to the bPAC S27A mutant (bPAC(S27A)-K)(n = 13, 5 cultures). **c** Light sensitivity. **d** Latency determined as the time needed to reach the signal onset from light stimulation. **e** On-kinetics determined as the slope measured from the signal onset to half peak maximum. **f** Current density as normalized photocurrents (current amplitude divided by cell capacitance) at 0.47 mJ·mm^{-2}. **g** Action spectrum of split-bPAC-K, split-TpPAC-K and bReaChES at constant photon exposure (n = 9, 4 cultures). Error bars in all graphs represent SEM.

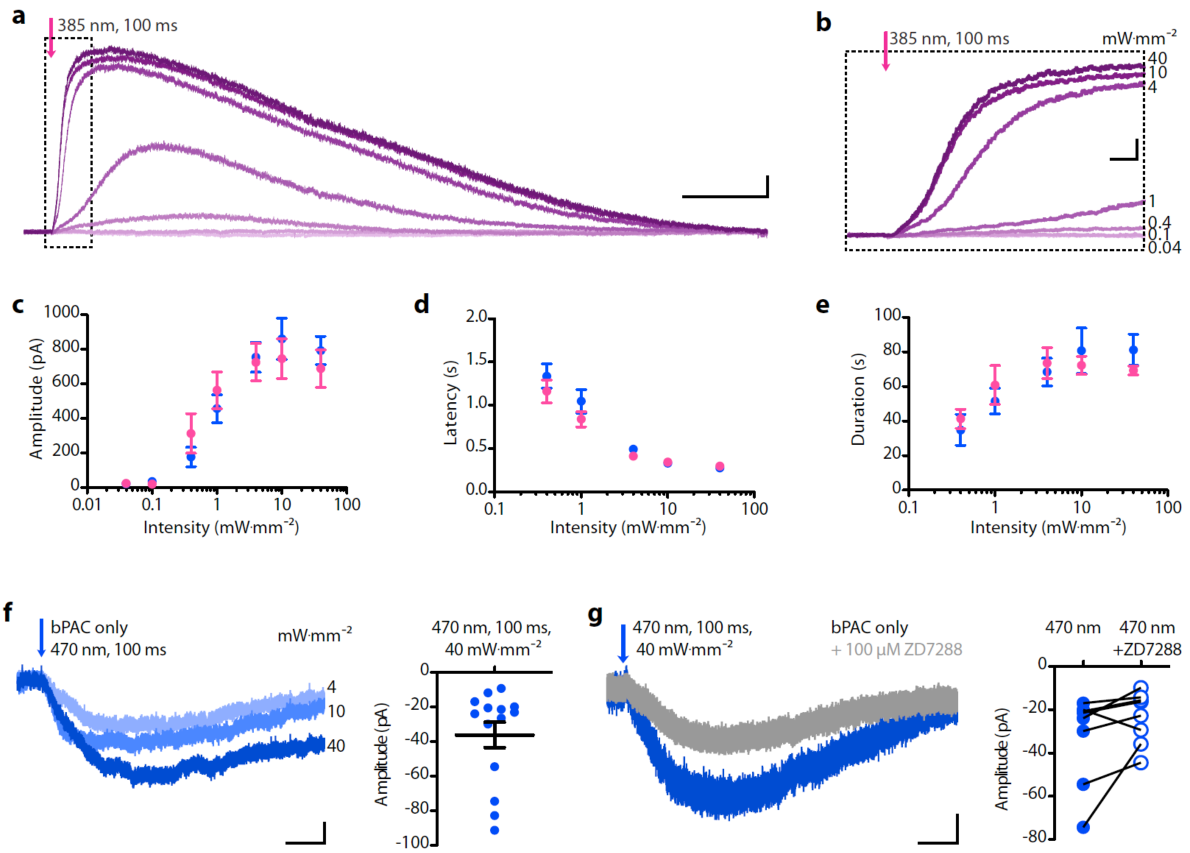

Supplementary Fig. 2: Characterization of bPAC-K in cultured neurons.
a Representative example of voltage-clamp recording from hippocampal neurons expressing split-bPAC-K. Light pulses were applied at different intensities for 100 ms. Scale bars: 10 s, 100 pA. **b** Inset from **a**, showing light intensity-dependent activation kinetics of the currents. Scale bars: 500 ms, 100 pA. **c** Peak current, **d** onset latency and **e** current duration of the SthK-mediated currents depend on the intensity of the activation light pulse for 385 nm (magenta) and 470 nm (blue) light (*n* = 8-10; 2 cultures). Latency and duration were not analyzed for intensities < 0.4 mW·mm^{-2}. **f** In cells expressing only bPAC-P2A-mCherry, 100 ms of 470 nm light evoked weak depolarizing somatic currents. The average current amplitude elicited by a 100 ms illumination with 470 nm light at 40 mW·mm^{-2} was -36.1±7.4 pA (*n* = 14). Scale bars: 10 s, 20 pA. **g** bPAC-evoked depolarization was partially sensitive to ZD7288 (100 µM), a blocker of hyperpolarization-activated cyclic nucleotide-gated (HCN)-channels (*n* = 8; *p* = 0.1, two-tailed paired *t*-test). Scale bars: 10 s, 20 pA. Error bars in all graphs represent SEM.

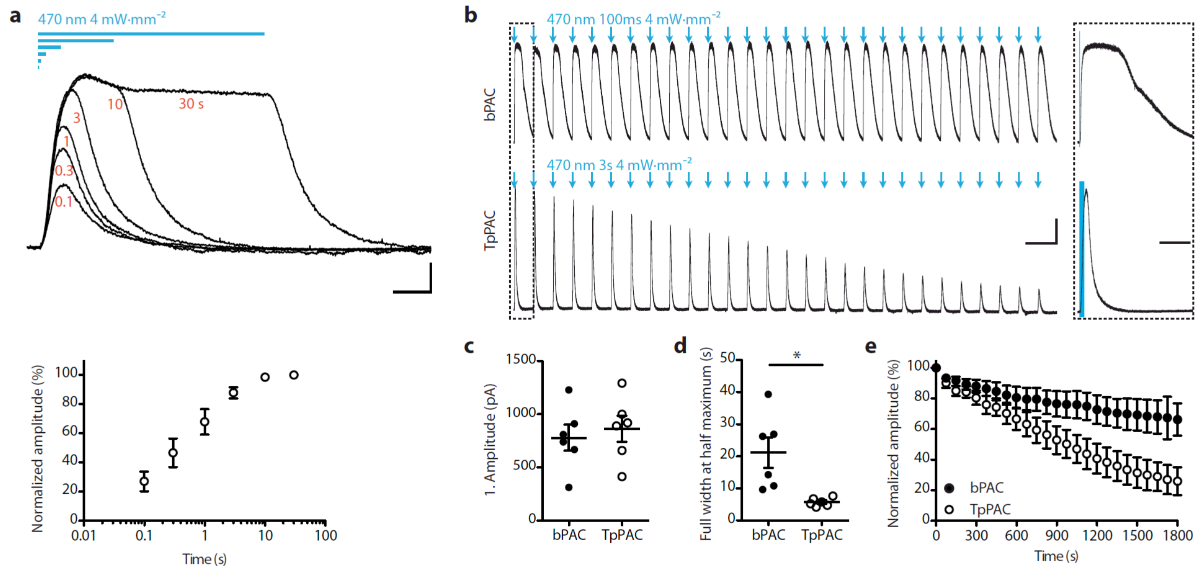

Supplementary Fig. 3: Characterization of split-TpPAC-K currents in cultured hippocampal neurons.

a Split-TpPAC-K showed illumination duration-dependent currents (*n* = 3-7, 2 cultures), with lower light sensitivity compared to split-bPAC-K (compare Fig. 3 **e**). Scale bars: 5 s, 200 pA. **b** Repetitive activation of split-bPAC-K and split-TpPAC-K. Neurons were illuminated at an interval of 75 s with 470 nm at 4 mW·mm^{-2} for 100 ms for bPAC, or 3 s for TpPAC. The longer illumination time was chosen to compensate for the lower light sensitivity of TpPAC. Inset shows currents elicited by the first light flash, with blue bars representing duration of the light flash. Scale bars: 120 s, 200 pA (overview trace), 20 s (inset). **c** Photocurrents evoked by the first light flash had similar amplitudes in split-bPAC-K and split-TpPAC-K-expressing cells (*n* = 6 for both groups, 1 culture; *p* = 0.64, unpaired two-tailed *t*-test). **d** TpPAC-driven SthK-currents had a significantly shorter full width at half maximum than bPAC-driven currents (*n* = 6 for both groups, 1 culture; *p* = 0.02, unpaired two-tailed *t*-test with Welch's correction). **e** Split-TpPAC-K currents showed -74 ± 9 % reduction in amplitude compared to -34 ± 10 % for split-bPAC-K currents after 24 repetitions within 30 min (*n* = 5 for both groups, 1 culture). Error bars in all graphs represent SEM.

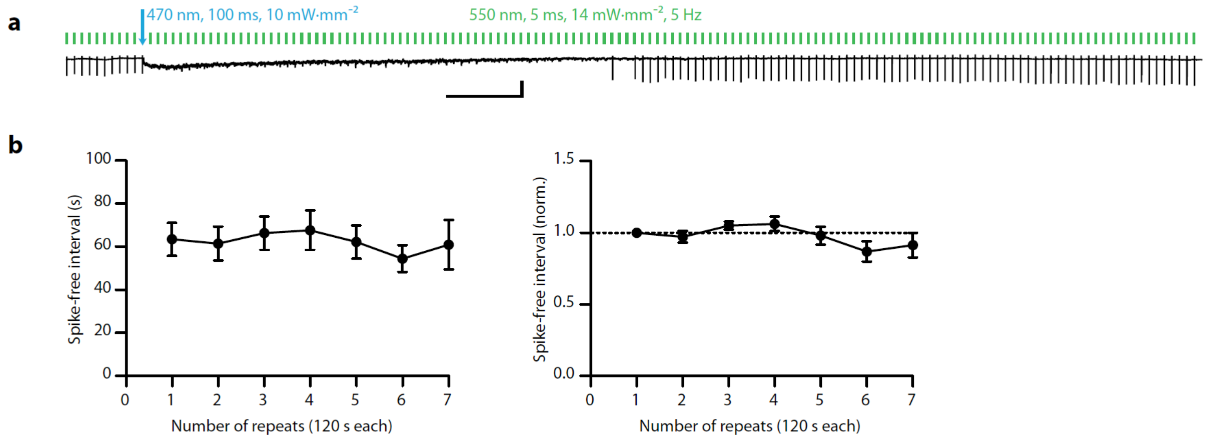

Supplementary Fig. 4: Stable, repetitive silencing by PAC-K activation in cell-attached recordings from neurons coexpressing split-bPAC-K and bReaChES.
a Exemplary trace of a cell-attached recording in a dual-color optogenetic excitation/inhibition experiment. Scale bars: 10 s, 100 pA. **b** Suppression of bReaChES-triggered spikes by split-bPAC-K activation lasted for 63 ± 8 s and remained largely stable over several repeats (*n* = 7, 2 cultures). Error bars represent SEM.

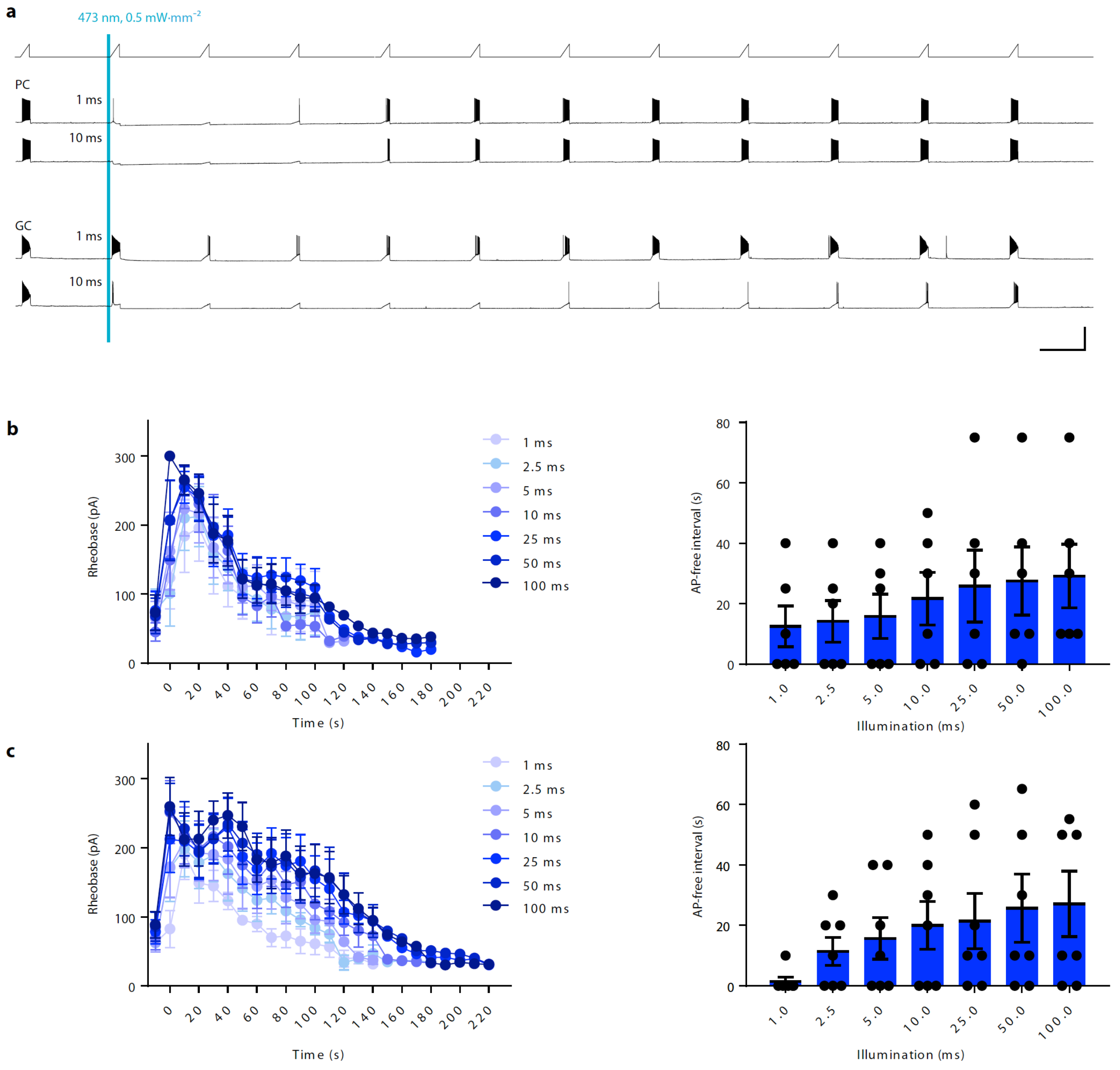

Supplementary Fig. 5: Silencing of AP-firing in different neuronal cell types in hippocampal slices using fused-bPAC-K.

a Representative examples of light-evoked inhibition of firing of a hippocampal CA1 pyramidal cell (PC) and a dentate gyrus granule cell (GC), each with two different durations of the light pulse (1 or 10 ms). Scale bars: 5 s, 500 pA or 100 mV, respectively. **b, c** Quantification of the silencing efficacy as increase of the rheobase over time after stimulation, for different durations of illumination (different blue colors, see legend, left panel). Right panel shows the average time period in which no firing was observed to ramp stimulation. Data shown for pyramidal cells (n = 6, 6 animals) in **b** and granule cells (n = 7, 6 animals) in **c**. Error bars in all graphs represent SEM.

Supplementary Table 1: Electrophysiological parameters of cultured hippocampal neurons expressing PAC-K

	uninfected (n=25)	split-PAC-K (n=29)	fused-PAC-K (n=21)	statistics
Capacitance	81.0 ± 6.0 pF	81.1 ± 4.5 pF	67.9 ± 4.1 pF	p = 0.13 (One-way ANOVA with Bonferroni's multiple comparison test)
Input resistance	158.3 ± 13.7 MΩ	166.5 ± 13.9 MΩ	142.6 ± 17.5 MΩ	p = 0.25 (Kruskal-Wallis test with Dunn's multiple comparison test)
Resting membrane voltage	-57.4 ± 1.2 mV	-58.3 ± 1.2 mV	-54.9 ± 1.5 mV	p = 0.22 (Kruskal-Wallis test with Dunn's multiple comparison test)

Basic electrophysiological parameters of cultured hippocampal neurons were not altered by viral expression of either split- or fused-bPAC-K (Multiple comparison tests: Capacitance: one-way ANOVA with Bonferroni's post-hoc test; Input resistance and resting membrane voltage: Kruskal-Wallis test with Dunn's post-hoc test; *n* = 21-29, cells from 2 cultures). Values are given as mean ± SEM.

Supplementary Table 2: Description of the used constructs

Expression cassette	Experiment	Fig.	Construct type	PAC	Plasmid backbone	Promoter
SthK-P2A-bPAC-mCherry	Characterization in ND7/23 cells	Fig. 1b, c, e-h Supplementary Fig. 1a-g	split-PAC-K	bPACwt	pCS2	CMV
bPAC-SthK-T2A-mCherry	Characterization in ND7/23 cells	Supplementary Fig. 1 d-g	fused-PAC-K	bPAC(S27A)	pCS2	CMV
bPAC-SthK	Imaging of cAMP levels in HEK 293 cells	Fig. 1d	fused-PAC-K	bPAC(S27A)	pcDNA3	CMV
SthK-P2A-bPAC-mCherry	Cardiomyocytes	Fig. 2	split-PAC-K	bPACwt	pAdeno	CMV
SthK-P2A-bPAC-mCherry	Hippocampal cell cultures	Fig. 3 Supplementary Figs. 2-4	split-PAC-K	bPACwt	pAAV-CW3SL	CaMKIIα
	Recordings on hippocampal slices	Fig. 5a-f				
	In vivo silicon probe recordings	Fig. 6a-d				
SthK-P2A-TpPAC-mCherry	Hippocampal cell cultures	Supplementary Fig. 3	split-PAC-K	TpPAC	pAAV-CW3SL	CaMKIIα
FLEX(SthK-P2A-bPAC-mCherry)	Recordings on hippocampal slices	Fig. 5c-e	split-PAC-K	bPACwt	pAAV-CW3SL	hSyn
bPAC-SthK-T2A-mCherry	Recordings on hippocampal slices	Fig. 5g, h Supplementary Fig. 5	fused-PAC-K	bPAC(S27A)	pAAV	hSyn
	In vivo Ca^{2+} imaging	Fig. 6e-h				
UAS:bPAC-Sthk-tagRFPt	Movement inhibition in zebrafish	Fig. 7	fused-PAC-K	bPACwt	pTol2/14XUAS	

Overview of the constructs used for the individual experiments. Split-PAC-K refers to the cleavable version with a P2A sequence between the sequences encoding the PAC and the SthK channel. Fused-PAC-K refers to constructs expressing the cyclase and the ion channel as a single fusion protein. CaMKIIα: calmodulin-dependent protein kinase II-alpha promoter. hSyn: human synapsin promoter. CMV: cytomegalovirus promoter. UAS: upstream activation sequence.
